# Orbital Compartment Syndrome Following Frontoethmoidal Mucopyocele: A Case Report

**DOI:** 10.1155/crop/8863514

**Published:** 2025-12-21

**Authors:** Pablo González-De-Los-Mártires, Gonzalo Guerrero-Pérez, Iñigo Salmerón-Garmendia, Amaia Garin-Balerdi, Beatriz Elso-Echeverría, Huban Atilla

**Affiliations:** ^1^ Ophthalmology Department, Fundación Hospital de Calahorra, Calahorra, La Rioja, Spain; ^2^ Ophthalmology Department, Complejo Hospitalario de Navarra, Pamplona, Navarra, Spain, pamplona.es; ^3^ Otorhinolaryngology Department, Hospital Universitario de Cruces, Barakaldo, País Vasco, Spain; ^4^ Otorhinolaryngology Department, Complejo Hospitalario de Navarra, Pamplona, Navarra, Spain, pamplona.es

**Keywords:** case report, endoscopic sinus surgery, mucopyocele, orbital compartmental syndrome, rhinosinusitis complications

## Abstract

**Background:**

The objective of this study is to describe a rare case of orbital compartment syndrome (OCS) following complicated frontoethmoidal mucopyoceles.

**Case Presentation:**

This report involves a 72‐year‐old woman with prior functional endoscopic sinus surgery who presented with acute painful proptosis, tense eyelid swelling, and ophthalmoplegia in her left eye. Imaging revealed infected mucoceles (mucopyoceles) in the left frontal and ethmoidal sinuses with intraorbital extension. Based on clinical and radiological findings, OCS was diagnosed. Emergency management included immediate lateral canthotomy and cantholysis, followed by urgent endonasal drainage of the mucopurulent material. The patient made a full recovery, with only transient eyelid numbness.

**Conclusion:**

This case underscores the importance of prompt recognition and timely coordinated intervention in OCS to prevent irreversible visual loss and intracranial extension.

## 1. Introduction

Chronic inflammation of the paranasal sinuses may lead to the development of nasal polyps and mucoceles, which are cavities filled with mucus due to obstructed sinus drainage [1]. These lesions most frequently involve the frontal and frontoethmoidal sinuses and typically present during middle to late adulthood, with no evident sex predilection [1]. The underlying pathogenesis is multifactorial, with contributing factors including trauma, chronic rhinosinusitis, neoplastic processes, prior sinonasal surgery, and allergic conditions [1, 2].

Postsurgical mucocele formation constitutes a recognized complication of functional endoscopic sinus surgery (FESS), with longitudinal studies reporting incidence rates of up to 13% [3]. Mucoceles may develop months to years after surgery due to ostial obstruction, scarring, or altered mucociliary clearance [4].

As mucoceles enlarge, they may induce pressure‐related erosion of adjacent bony structures and extend into contiguous anatomical spaces, most notably the orbit [1]. Subsequent infection may convert a mucocele into a mucopyocele, characterized by a more aggressive clinical course and an increased risk of complications, including orbital compartment syndrome (OCS) [5].

This case illustrates a rare acute complication of an infected postsurgical mucocele, emphasizing the risk of compressive optic neuropathy and intracranial extension and the critical importance of early recognition and timely surgical intervention.

## 2. Case Presentation

A 72‐year‐old woman presented to the emergency department with a 2‐day history of progressive redness in the left upper eyelid, which had acutely worsened during the hour preceding her arrival. This was accompanied by severe ocular pain, pronounced eyelid swelling, and marked conjunctival chemosis.

Her medical history included a FESS performed 3 years earlier to address chronic sinusitis and sinonasal polyposis. No additional ophthalmologic or medical records were available, and she had not undergone routine postoperative follow‐up, which may have contributed to the delayed detection of mucocele formation. The patient had no significant comorbidities.

**Figure 1:**
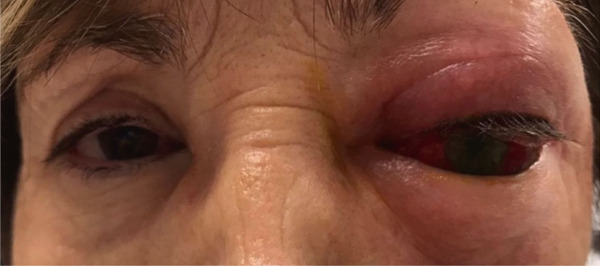
Left eye condition in the emergency room. Swelling and redness of the upper eyelid associated with prominent proptosis, chemosis, and inferolateral dystopia.

At presentation, visual acuity in the left eye had deteriorated from 8/10 to hand motion perception. Clinical examination revealed restricted extraocular motility in all directions, a relative afferent pupillary defect, and exophthalmos with inferolateral displacement of the eyeball (Figure 1). Slit‐lamp examination showed corneal edema, and intraocular pressure was recorded at 52 mmHg. Due to the severity of findings, an emergent left lateral canthotomy and cantholysis were performed. In addition, topical and oral hypotensive agents (timolol–brimonidine eye drops and oral acetazolamide 250 mg) were administered along with intravenous analgesia (dexketoprofen 25 mg and meperidine 50 mg).

A subsequent blood test indicated leukocytosis with neutrophilia and an elevated C‐reactive protein level. Consequently, intravenous broad‐spectrum antibiotics were administered. Orbital and sinus computed tomography (CT) scan revealed left frontal and ethmoidal mucoceles extending into the extraconal intraorbital space (Figure 2a,b). The abrupt onset of exophthalmos was attributed to the enlargement of the aforementioned mucoceles, driven by inflammatory and infectious changes involving the postseptal and orbital apex. Otolaryngologic evaluation revealed postsurgical alterations, with partially healed ethmoidal cells and minimal purulent discharge observed on nasosinusal fibroscopy.

Figure 2Paranasal and orbital CT at the emergency room. (a) Coronal projection: a large mucopurulent collection in the ethmoidal and frontal sinuses, displacing the left eyeball outward. (b) Axial projection: an enlarged frontoethmoidal mucopyocele, resulting in significant proptosis.

**Figure:**
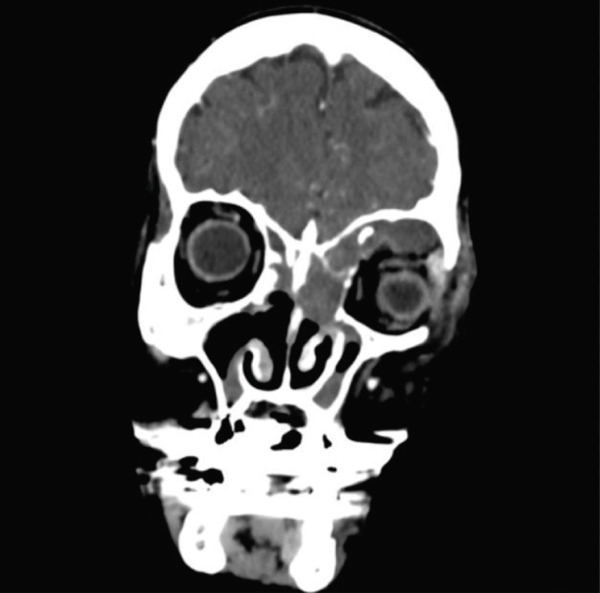
(a)

**Figure:**
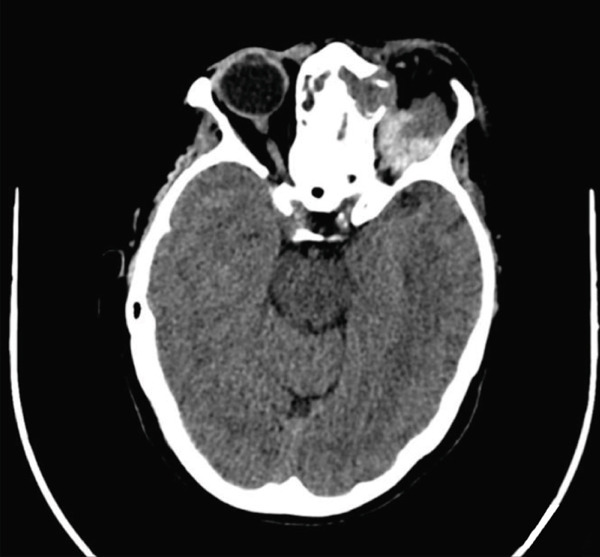
(b)

Based on the clinical and radiological findings, a diagnosis of OCS secondary to infected frontoethmoidal mucoceles was established. This entity is distinguished from other etiologies of red eye by the presence of retrobulbar pain, proptosis, ophthalmoplegia, decreased visual acuity, and elevated intraocular pressure.

The patient was urgently admitted for a new FESS under general anesthesia. Owing to residual eyelid tightness, the initial canthotomy and cantholysis were extended. The procedure involved opening the middle meatus, performing the marsupialization of the mucopyoceles via the frontal recess to facilitate drainage of the mucopurulent material, which led to immediate postoperative improvement. Microbiological analysis of intraoperative specimens yielded *Streptococcus pyogenes* and *Moraxella catarrhalis*, both susceptible to amoxicillin–clavulanate.

The patient remained hospitalized for 1 week, receiving intensive intravenous antibiotic and corticosteroid treatment (amoxicillin–clavulanate 1 g every 8 h and methylprednisolone 40 mg every 12 h), in addition to intranasal corticosteroid (beclomethasone 500 mcg every 12 h). Two weeks postoperatively, proptosis had resolved, allowing complete eyelid closure. Nonetheless, residual erythema, difficulty with eyelid opening, and paresthesia in the left supraorbital region persisted. Visual acuity improved to 7/10, despite the presence of diffuse keratitis, which responded favorably to ocular lubricants. Intraocular pressure decreased to 17 mmHg, and ocular motility was preserved. Optical coherence tomography of the optic nerve revealed no structural abnormalities.

**Figure 3:**
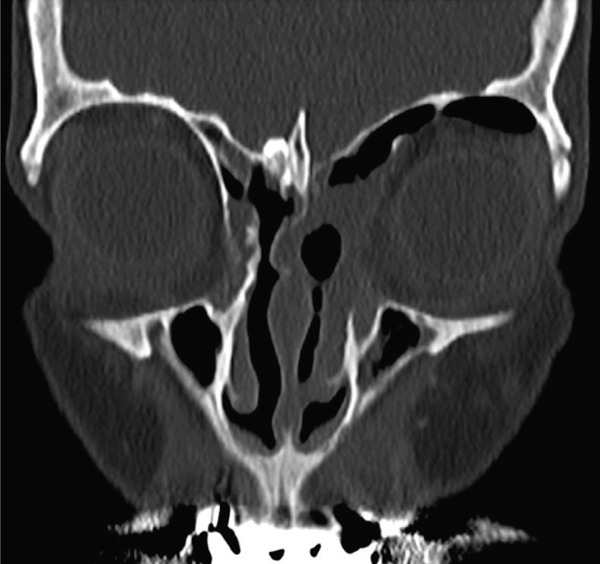
One‐year postoperative paranasal and orbital CT scan. Coronal projection: continuous opening of the medial meatus and ethmoidal sinus, along with pneumatization of frontoethmoidal mucoceles. The globe is in normal position.

At 1‐year follow‐up, serial CT scans performed at 6‐month intervals demonstrated no evidence of restenosis, recurrence, or the formation of new mucoceles (Figure 3).

## 3. Discussion

Frontal and frontoethmoidal mucopyoceles frequently induce anterior displacement of the globe due to their mass effect, a phenomenon seldom encountered in those originating from the posterior ethmoid or sphenoid sinuses [5, 6]. Orbital involvement typically occurs via direct extension through defects in the lamina papyracea, rather than through hematogenous spread [7]. Consequently, orbital progression and risk of OCS are more commonly associated with frontal and frontoethmoidal mucopyoceles [5].

In this report, the patient exhibited hallmark features of OCS, including acute painful proptosis, ophthalmoplegia, relative afferent pupillary defect, and significantly elevated intraocular pressure [8]. While the diagnosis is primarily clinical, radiological imaging, especially noncontrast CT, supports assessment of bony structures and sinus opacification [1, 6]. Although MRI offers enhanced soft tissue resolution, it was deemed unnecessary in this case due to the previous history of sinonasal surgery.

Initial management of OCS necessitates emergent orbital decompression, typically via lateral canthotomy and cantholysis, combined with broad‐spectrum intravenous antibiotics [9]. Definitive treatment requires surgical drainage of the mucopyocele, which can be achieved through endoscopic or external approaches [9]. Although irreversible optic nerve injury is commonly associated with ischemic intervals exceeding 2–4 h, visual function may be preserved if intervention occurs within 48 h and axonal integrity is maintained [9, 10].

Despite delayed presentation, substantial visual recovery was achieved, emphasizing that timely intervention even near the margin of the recommended therapeutic window can preserve vision. This case also highlights broader implications for postoperative management of high‐risk patients. Routine long‐term follow‐up, including periodic endoscopic evaluation and imaging after FESS, may allow early detection of asymptomatic mucoceles, preventing severe complications such as OCS [3, 4, 11].

The combined medical and surgical approach led to full resolution of OCS without permanent optic nerve damage, although transient ptosis and periorbital numbness persisted, likely from compression‐related neuropraxia [1, 9]. Antimicrobial therapy controlled the infection, while adjunctive corticosteroids accelerated inflammation resolution. However, their use should follow microbiological confirmation to ensure safety [12]. Endoscopic marsupialization effectively re‐established sinus drainage and relieved orbital pressure, providing a minimally invasive option with superior visualization, lower recurrence rates, and favorable cosmetic outcomes [9, 13].

## 4. Conclusion

Mucopyoceles, particularly those of the frontal and ethmoid sinuses, may manifest with diverse clinical features, with orbital extension carrying a high risk of OCS. Prompt diagnosis based on clinical and radiological findings is essential. Management requires immediate orbital decompression and broad‐spectrum antibiotics, often complemented with corticosteroids after microbiological confirmation. Endoscopic surgery remains the preferred definitive treatment, as it restores sinus drainage while minimizing morbidity. Urgent, coordinated care is critical to prevent vision loss and intracranial complications, and long‐term follow‐up is recommended to identify recurrence or sequelae. Routine post‐FESS monitoring in high‐risk patients should be considered to prevent delayed mucocele formation and associated complications.

## Ethics Statement

For this type of study, ethical approval is not required. The article is written according to the World Medical Association Declaration of Helsinki.

## Consent

No written consent has been obtained from the patients as there is no patient identifiable data included in this case report.

## Disclosure

All authors attest that they meet the current ICMJE criteria for authorship.

## Conflicts of Interest

The authors declare no conflicts of interest.

## Author Contributions

P.G.D.L.M. is responsible for design, concept, intellectual content, manuscript preparation, literature search, and data acquisition/analysis. G.G‐P., I.S‐G., A.G‐B., and B.E‐E., are responsible for manuscript proofreading, editing and review.

## Funding

No funding was received for this manuscript.

## Data Availability

The data that support the findings of this study are available from the corresponding author upon reasonable request.
